# Let’s not be indifferent about robots: Neutral ratings on bipolar measures mask ambivalence in attitudes towards robots

**DOI:** 10.1371/journal.pone.0244697

**Published:** 2021-01-13

**Authors:** Julia G. Stapels, Friederike Eyssel

**Affiliations:** Center for Cognitive Interaction Technology, Department of Psychology, Bielefeld University, Bielefeld, Germany; University of Auckland, NEW ZEALAND

## Abstract

Ambivalence, the simultaneous experience of both positive and negative feelings about one and the same attitude object, has been investigated within psychological attitude research for decades. Ambivalence is interpreted as an attitudinal conflict with distinct affective, behavioral, and cognitive consequences. In social psychological research, it has been shown that ambivalence is sometimes confused with neutrality due to the use of measures that cannot distinguish between neutrality and ambivalence. Likewise, in social robotics research the attitudes of users are often characterized as neutral. We assume that this is due to the fact that existing research regarding attitudes towards robots lacks the opportunity to measure ambivalence. In the current experiment (N = 45), we show that a neutral and a robot stimulus were evaluated equivalently when using a bipolar item, but evaluations differed greatly regarding self-reported ambivalence and arousal. This points to attitudes towards robots being in fact highly ambivalent, although they might appear neutral depending on the measurement method. To gain valid insights into people’s attitudes towards robots, positive and negative evaluations of robots should be measured separately, providing participants with measures to express evaluative conflict instead of administering bipolar items. Acknowledging the role of ambivalence in attitude research focusing on robots has the potential to deepen our understanding of users’ attitudes and their potential evaluative conflicts, and thus improve predictions of behavior from attitudes towards robots.

## Introduction

Would you like to have a social robot at home? You may have mixed feelings about an artificial intelligence system in your private space. On the one hand, owning a robot may sound promising. Social robots can be entertaining, while they might also assist with chores or serve as companions. On the other hand, such technology gives rise to privacy and security concerns which are commonly associated with mobile, cloud-connected devices making use of a camera or a microphone [1]. You might feel both positively and negatively about having a robot at home, and this makes the question of ultimately buying a robot discomforting and difficult to answer. It might be that you find yourself in a state of evaluative conflict. That is, you are experiencing ambivalence.

Now think of a simple office supply tool such as a stapler. You might also not have a clearly positive or negative opinion about staplers, since staplers are somewhat useful, but usually do not evoke strong feelings. Staplers are evaluated as neutral, not as conflicting [2]. While you might feel torn between strong positive and negative evaluations concerning robots, you might experience the absence of strong evaluations concerning staplers. When asked whether your attitude concerning both objects is either positive or negative, you will likely say both times that it is neither positive nor negative, but somewhere in between. However, this example shows that a rating between positive and negative can imply different states of mind: ambivalence (i.e., strong competing evaluations), or neutrality (i.e., the absence of strong evaluations).

In this work, we extend the generalizability of the ambivalence construct to the domain of robotics. We propose that robots represent a source of attitudinal ambivalence, and that measuring ambivalence towards robots and understanding the psychological antecedents and consequences of ambivalence advances research on attitudes towards robots.

## Related work

### Ambivalence in psychological attitude research

In social psychology, attitudes are defined as all evaluations about one object of thought [3]. Attitudes have cognitive, affective and behavioral components, guiding information processing and behavior [4]. When these evaluations are simultaneously experienced as both positive and negative regarding one and the same attitude object, the attitude is said to be ambivalent [4–6]. Ambivalence is an evaluative conflict, a phenomenon which has been investigated in various domains, for instance, with regard to food choices [7], online transactions [8], or artificial intelligence [9]. When confronted with highly ambivalent attitude objects, people take longer to evaluate them [10], report negative affect and show physiological arousal [11].

**Fig 1:**
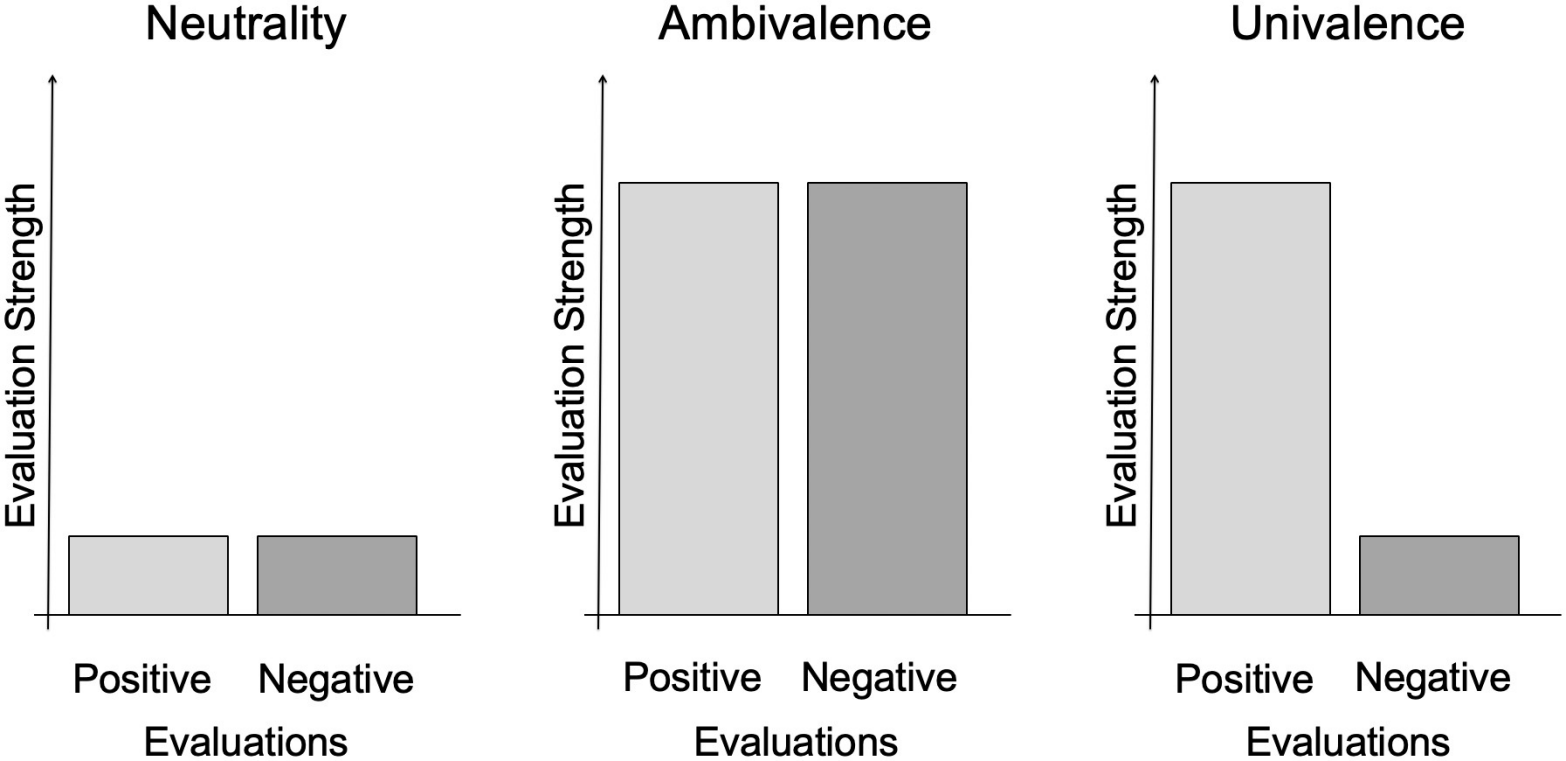
Strengths of positive and negative evaluations for neutral, ambivalent, and univalent positive attitudes.

In order to analyze ambivalence, the construct has to be distinguished from conceptually distinct constructs such as neutrality, ambiguity [12], and cognitive dissonance [11]. Although on the surface these constructs may seem similar, there are important differences to be mentioned: First, ambivalence is defined as the state of having conflicting positive and negative evaluations towards an attitude object [5]. In contrast, *neutrality* refers to an attitude lacking strong positive or negative evaluations [5]. Both ambivalent and neutral attitudes are neither clearly positive nor negative, but whereas neutral attitudes are neither positive nor negative, ambivalent attitudes are positive and negative at the same time. In Fig 1 we provide an illustration of the differences between neutral, ambivalent, and univalent attitudes.

Second, *ambiguity* results from a lack of information which makes it difficult to form an attitude towards an object [13]. Think of a simple scenario: Participants in a research study are asked to evaluate a robot based on one picture only. Given the limited amount of information available, the participants might not have much to say about the attitude object. When in a state of ambiguity, people have not yet formed evaluations which could potentially cause a conflict. In contrast, ambivalence is accompanied by unpleasant feelings such as arousal and feelings of conflict which may influence subsequent decision making. This is neither the case for neutrality nor ambiguity [14]. Thus, ambivalence is a state of evaluative conflict, while neutrality and ambiguity are not.

Third, ambivalence is often compared to *cognitive dissonance*, a state of psychological discomfort emerging from competing or incompatible cognitions and behavior [15]. Motivations towards reducing dissonance and ambivalence may stem from similar motives, namely reducing inconsistencies. However, cognitive dissonance is described as an experience associated with conflicting cognitions or a conflict between cognitions and behavior *after* a decision is made, whereas ambivalence characterizes a conflict between cognitions *before* a decision is made [16]. Further, some authors see the distinction as a methodological one, as in dissonance studies inconsistency is manipulated on an absolute level (it is either present or absent) and the psychological consequences are measured. Ambivalence research, however, provides opportunities to manipulate and measure the magnitude of inconsistencies [16]. Distinguishing ambivalence from cognitive dissonance brings the benefit of investigating evaluative conflict as an independent *or* dependent variable, using widely validated manipulation and measurement methods. Further, based on indicators of ambivalence, empirically based predictions for affective, behavioral and cognitive consequences can be made [6]. The expected consequences of ambivalence are more specific than those of general dissonance that is either present or absent and promotes a general motivation towards reduction. In sum, ambivalent attitudes reflect the co-existence of both positive and negative evaluations, whereas the magnitude of ambivalence can vary depending on the content of the evaluations.

### Ambivalence in attitudes towards robots

Although people tend to be interested in the topic of robots and may even be keen to meet one, robots are still not yet prevalent in households. This contradiction is likely caused by manifold reasons, such as users’ control beliefs, and social and personal norms [17]. Ambivalence might additionally influence the decision making process due to ambivalent attitudes and the desire of users to resolve attitudinal conflicts before committing to have a robot at home. Users may be avoiding an object that causes them discomfort in the long term. Previous research has suggested that users are aware of many important positive as well as negative aspects concerning having a robot at home.

#### Positive and negative aspects of social robots in the home context

Earlier works have elucidated the specific content of positive and negative evaluations regarding attitudes towards robots [1, 18–21]. To illustrate, when deciding whether to have a social robot at home, user decisions might be influenced by experiences with the technological devices they themselves currently use. Users expect these devices to help them with everyday organizational tasks, such as managing their calendar and to-do lists, controlling their smart home or providing information on demand [18]. Recently developed social robots (e.g., Vector by Anki [22]) offer functions similar to those performed by smart home devices (e.g., serving as simple conversational agents or searching for information on the internet), while additionally being mobile and capable of perceiving their environment. Users anticipate social robots to be at least as capable as smart home devices, due to their ability to support them by completing household chores or tasks related to food preparation or transportation [20].

To some degree, social robots are expected to be highly useful in everyday life, though they may also be perceived as a threat: Devices which perform assistive functions in the homes of users (e.g., voice assistants) have already been heavily criticized for various security and privacy issues [23]. Robots could prove just as problematic. Users might for example feel observed because robot platforms have the capacity to collect vast amounts of auditory and visual data in user’s homes [19], which could then be misused by developers or hackers [1]. Furthermore, user privacy and security concerns are not necessarily objective but instead likely to be negatively biased, since expectations towards robots have to some extent been shaped by negative portrayals in science fiction movies [20, 24, 25]. Another concern commonly voiced by users is the fear of dependence upon increasingly autonomous robots, whereby users could become less and less autonomous themselves [21].

As such, previous research has discussed both positive and negative attributes concerning social robots within home environments. Whereas robots are deemed useful, they likewise bear various risks, which might give rise to an evaluative conflict that results in ambivalent attitudes towards robots.

#### Indications of attitudinal conflict in social robotics research

To date, few researchers have directly addressed the notion of ambivalence in attitudes towards robots. MacDorman and colleagues [26] have discussed ambivalence in the context of robots as an inherent conflict between technological progress and the fear that robots might one day become too powerful and harm humans, as portrayed in many science fiction movies [27].

Further, empirical studies have found evidence for ambivalent attitudes using mixed-methods approaches [20, 21, 28]. Frennert and colleagues [28] have, for instance, investigated expectations of elderly participants regarding assistive domestic robots. Qualitative results revealed that participants felt torn between the potential benefits of living with domestic robots and their fears of becoming dependent on a machine or being alienated from human contact. The authors extended these findings in a case study [21] which provided elderly users with a domestic robot in their home for three weeks. In the qualitative responses, participants also reported ambivalence regarding the benefits (e.g., assistance in everyday life) and disadvantages (e. g., concerns around seeming helpless or fragile) of having a domestic robot at home [21].

However, conflicting evaluations concerning robots are not restricted to elderly users: To illustrate, Horstmann and Krämer [20] conducted semi-structured interviews which revealed that university students on the one hand expected social robots to be highly useful, while also having the science-fiction inspired fear of humanity being threatened by conscious robots. Thus, the reviewed results based on qualitative data indicated that users hold mixed evaluations that are commonly not captured by quantitative measures. The qualitative data then suggested that user attitudes towards robots were positive and negative at the same time.

Similarly, quantitative results by Syrdal and colleagues [29] implied that attitudes towards robots might be ambivalent. Upon developing the multidimensional Frankenstein Syndrome Questionnaire (FSQ), they measured attitudes towards robots using the subscales *General Negative Attitudes towards Robots*, *General Positive Attitudes towards Robots*, *Principal Objections to Humanoid Robots*, *Trust in Robot Creators* and *Interpersonal Fears*. The first two subscales serve to measure positive and negative attitudes separately, demonstrating that they may be partially independent. In a validation study with Japanese (*n* = 1000) and European participants (*n* = 146), the subscales for positive and negative attitudes correlated only weakly with each other when considering all participants (*r* (1144) = .21) and not at all in the Japanese sample (*r* (998) = .01). This is in line with attitude research by Cacioppo and colleagues [30] showing that positivity and negativity are partly independent dimensions. Hence, strong positive attitudes do not necessarily imply the absence of negative attitudes.

As the studies imply, many people seem to acknowledge both positive and negative aspects regarding robots, and possibly experience ambivalence. As such, there seems to be reason to believe that attitudes towards robots are indeed ambivalent. While the existing research further underlines the relevance of the notion of ambivalence for robotics research, quantitative research on ambivalent attitudes towards robots might thus far have been neglected because researchers often use bipolar measures, which may produce similar results for neutral and ambivalent attitudes.

#### Mistaking ambivalence for neutrality

Attitudes are often investigated using bipolar items. For instance, in empirical research using surveys, participants are likely asked to rate their attitudes towards robots on a scale from *very negative* to *very positive* [31]. One of the largest studies on attitudes towards robots featuring over 80,000 participants, the 2017 European Barometer survey [32], investigated attitudes towards robots using bipolar scales to collect responses. In the survey, participants were asked “Generally speaking, do you have a very positive, fairly positive, fairly negative or very negative view of robots?”, as well as how comfortable they were about “things that could be done by robots” such as performing medical operations or assisting at work, on a scale from 1 (totally uncomfortable) to 10 (totally comfortable)—giving participants no possibility to express conflict. Overall, the mean attitude was not far from the scale midpoint [31]. Importantly, a supposedly neutral rating could mean different things: For one, it could mean that a person is indifferent about the respective attitude object (in this case, robots), implying a weak attitude. However, a mean score not far from the scale midpoint could also indicate coexisting strong positive *and* strong negative attitudes about robots, resulting in the experience of ambivalence, and reflecting a strong attitude [14, 33, 34]. While previous research has assumed that ambivalent attitudes are only weakly associated with behavior, a recent meta-analysis revealed that this is only the case when people perceive themselves as having a profound knowledge of the concerning attitude object. When people perceive themselves as less knowledgeable about the attitude object however, ambivalent attitudes and univalent attitudes toward the attitude object turn out to be equally predictive of behavior [35]. This example shows that it is highly useful for research on attitudes towards robots to distinguish neutral attitudes from ambivalent attitudes in order to predict subsequent behavior.

Schneider and colleagues [14] have demonstrated empirically that neutrality and ambivalence are often confused when attitude objects are evaluated. Their experiment showed that neutral stimulus materials could evoke a wide range of evaluative conflict—moreover, conflict which could not be captured by bipolar scales. Participants evaluated 29 “neutral” pictures from the International Affective Picture System (IAPS). All pictures were evaluated as neutral on bipolar scales from *negative* to *positive* but varied widely concerning ambivalence. In addition, the experience of ambivalence was predictive of arousal. The authors conclude that bipolar measures alone can not indicate neutrality, since they do not reflect affective responses. Therefore, using bipolar measures makes it impossible to distinguish ambivalence from neutrality. By comparing bipolar measures with multi-dimensional measures in the current study we demonstrate that this distinction between ambivalence and neutrality is also relevant in robotics research.

## The present experiment

In this experiment, we employ similar measures as in Schneider and colleagues [14] to demonstrate empirically that attitudes towards robots might appear neutral when relying upon data gathered from bipolar measures alone, while these attitudes are in fact ambivalent. We do so by measuring the positive and negative aspects of attitudes towards robots separately and by giving participants the opportunity to express their experienced ambivalence and arousal in addition to bipolar valence ratings. The distinction between neutrality and ambivalence is practically relevant because of the negative affective consequences resulting from the experience of ambivalence, namely increased arousal. We hypothesize, that attitudes towards robots are ambivalent too, but can appear neutral on bipolar measures. For this experiment, we compared attitudes towards robots with attitudes towards staplers, as neutral attitude objects. We selected staplers as comparisons because staplers are perceived as neutral according to our pilot study (see Materials section). Our preregistered hypotheses were as follows (https://aspredicted.org/72nj4.pdf): Participant ratings of robots and staplers will not differ in valence measured using a bipolar item (H1); participants will report higher subjective ambivalence (H2), objective ambivalence (H3) and arousal (H4) towards robots than towards staplers.

## Method

### Pilot study

#### Participants, design and measures

Initially we conducted a preregistered (https://aspredicted.org/33u6p.pdf) pilot study with 39 participants (35 female, 4 male, *M*_Age_ = 22.38, *SD*_Age_ = 3.02) at Cologne University to identify robot-related and neutral stimuli that would be evaluated as equally neutral on a bipolar valence scale. We did not aim for an equal gender distribution, as the expected effect should be comparable in all genders (see [14]).

All participants were presented with six pictures featuring three robots (Robosapien X, Asimo, Myon) and three neutral items (stapler, paperclips, paper), as well as three words which were either robot-related or neutral (robot, stone, stapler; German: Roboter, Stein, Tacker). Neutral pictures were taken from the Open Affective Standardized Image Set (OASIS). In the original study, these pictures were rated as neutral in valence (i.e., mean valence rating between *M* = 3.99 (*SD* = 0.46) and *M* = 4.09 (*SD* = 0.51) on a 7-point scale) and low in arousal (i.e., between *M* = 1.84 (*SD* = 1.26) and *M* = 2.00 (*SD* = 1.34) on a 7-point scale) [2]. The term “stone” was selected as an additional neutral stimulus word from the Berlin Affective Word List [36].

#### Procedure

First, participants were presented with all words in a random order and were asked for their evaluation following each word on a 7-point Likert scale from 1 (negative) to 7 (positive). Following this, participants saw the six pictures in a randomized order and evaluated them using the same item. This research was approved by the Ethics Committee of Bielefeld University.

#### Results

The words “robot”, “stapler”, and “stone” were all evaluated near the midpoint of the bipolar scale (see Table 1). As preregistered, we used equivalence testing to determine whether the evaluations were statistically equivalent, since a conventional t-test is not adequate for determining the statistical equivalence of two means (see [37, 38]). For the words “robot” and “stapler”, the equivalence test was significant, *t*(38) = -1.812, *p* = 0.039, with equivalence bounds of -0.58 and 0.42 (on a raw scale). The null hypothesis test was not significant, *t*(38) = -0.259, *p* = 0.797. Based on the equivalence and null-hypothesis tests combined, we can conclude that the observed effect is statistically not different from zero and statistically equivalent to zero. That is, the words “robot” and “stapler” both appear neutral on a bipolar item assessing valence. For the words “robot” and “stone” the equivalence test was not significant *t*(38) = 1.382, *p* = .087, with the same equivalence bounds. The null hypothesis test was not significant either, *t*(38) = -0.727, *p* = 0.471. This indicates that the effect is not different from zero, but also not significantly equivalent to zero, probably due to the larger variance in the stone evaluations. We therefore selected the words “robot” and “stapler” as stimuli for the main study to demonstrate differences regarding evaluative conflict and arousal. As preregistered, we selected the words as stimuli over the pictures, since we aimed to examine participants’ general attitudes towards robots, unrelated to specific robot designs.

**Table 1:** Means and standard deviations for word and picture stimuli on a bipolar valence scale from 1 (negative) to 7 (positive).

	Stimuli	*M*	*SD*
**Words**	robot	4.03	1.22
	stapler	4.10	1.12
	stone	4.23	1.31
**Pictures**	Robosapien X	3.95	1.41
	Asimo	4.10	1.52
	Myon	3.87	1.45
	stapler	4.13	1.15
	paperclips	4.38	1.27
	paper	4.67	1.30

### Main experiment

#### Participants and design

Fifty participants were recruited on the campus of Bielefeld University and volunteered to participate in a computerized study on measurement methods. As preregistered, we excluded 5 datasets because they did not pass the self-report attention check at the end of the study. Forty-five complete datasets (35 female, 10 male, *M*_*age*_ = 22.04, *SD*_*age*_ = 3.11) were included in the analysis, as planned in an a priori power analysis of a one-tailed t-test for an effect size of *d* = 0.5, an alpha error probability of 5% and a Power of 95%. We did not explicitly aim for an equal gender distribution, as the expected effect should be comparable in all genders (see [14]). Most participants were students (42 students, 1 employee, 1 unemployed, 1 other) and all participants were at least fluent in German. Participants rated both stimuli (robots and staplers) using both bipolar valence scales as well as measures of ambivalence in a two-factorial within-subjects design. In addition, we assessed participants self-reported arousal as an affective consequence of ambivalence. We report how we determined our sample size, all data exclusions (if any), all manipulations, and all measures in the study or in the linked preregistrations.

#### Measures

As in [14], we used one of the Self-Assessment Manikin (SAM) scales by [39] to measure valence on a visual 9-point item reading “How do you appraise robots [staplers] in general?”, with 1 indicating *negative* and 9 indicating *positive*. In contrast to the pretest, we used a 9-point Likert response format in the main study, as a 9-point response format is commonly used for SAM scale designs.

To assess objective ambivalence, defined as the mere existence of opposing evaluations, two items assessed the positive and negative evaluations of the attitude objects separately (“When you think about the positive aspects of robots [staplers] and ignore the negative aspects, how positive is your evaluation of robots [staplers]?”, “When you think about the negative aspects of robots [staplers] and ignore the positive aspects, how negative is your evaluation of robots [staplers]?). We calculated a quantitative estimate of objective ambivalence which integrates positive and negative evaluations using a simple formula ((P + N)/2)—| P − N |) [4], with P representing the positive and N representing the negative evaluation. Low values in both P and N or a high rating on only one item result in small values for objective ambivalence, whereas high values in both P and N result in high values for objective ambivalence.

We used three items to assess subjective ambivalence using Likert scales, specifically conflicting thoughts and feelings, indecision and the experience of mixed feelings [40]. This measure gives participants the opportunity to express conflict arising from opposing evaluations and is predictive of negative affect as well as arousal. The items read: “To what extent do you have conflicting thoughts and feelings towards robots [staplers]?”, “To what extent do you feel indecisive about robots [staplers]?” and “To what extent do you experience mixed feelings concerning robots [staplers]?”. Responses were provided using a Likert scale ranging from 1 (not at all) to 9 (very much).

For the arousal rating, as in [14], we used one of the SAM scales on a visual 9-point item reading “How do you appraise robots [staplers] in general?”, with 1 indicating *boring* and 9 indicating *exciting* [39].

#### Procedure

Participants were instructed to evaluate objects in order to compare several question types with one another. After providing informed consent, they were presented with two example items from the SAM scale for valence and arousal [39]. In the main section of the study, participants first evaluated valence and arousal concerning “robots” and “staplers”. They then completed the measures for subjective ambivalence and objective ambivalence, respectively. We randomized the order of stimulus objects (that is whether “robots” or “staplers” were evaluated first) for both types of measures. After providing demographic information (sex, age, employment, German language skills), participants completed a self-report data quality check (“Should we use your data in the analysis?”), were thanked and dismissed. This research was approved by the Ethics Committee of Bielefeld University.

## Results

**Fig 2:**
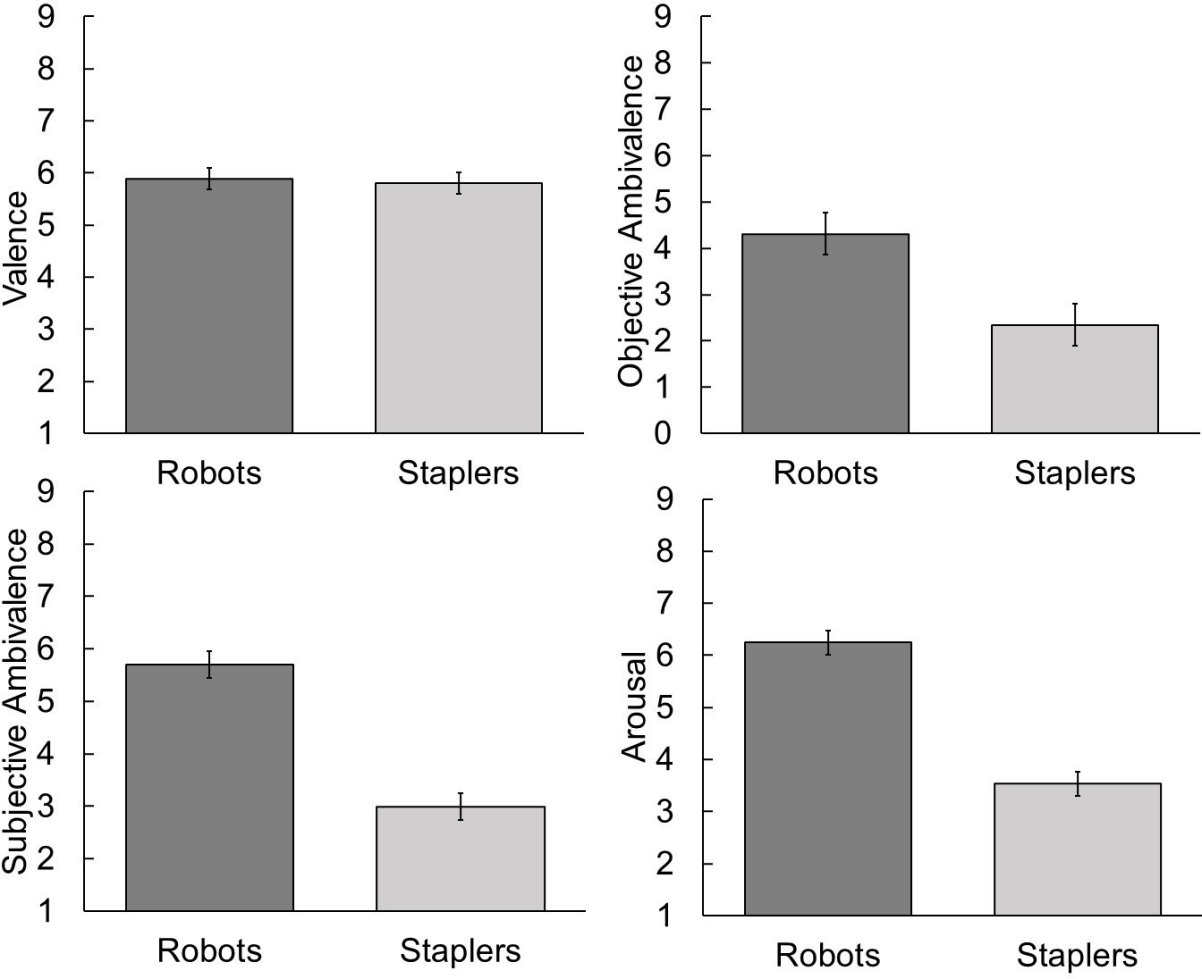
Mean valence, objective ambivalence, subjective ambivalence, and arousal per condition.

We used the statistical software R to conduct analyses. Results were in line with the four preregistered hypotheses. First, in order to test the equivalence of valence evaluations of robots (*M* = 5.89, *SD* = 1.54) and staplers (*M* = 5.80, *SD* = 1.44) on a bipolar scale (H1), we conducted two one sided t-tests in addition to testing the mean difference against zero (see [37]). The equivalence test was significant, *t*(44) = -3.41, *p* < .001, with equivalence bounds of -1 and 1 (on a raw scale). The null hypothesis test was not significant, *t*(44) = 0.34, *p* = 0.737. Based on the equivalence and null-hypothesis tests combined, we can conclude that the observed difference in valence evaluations between robots and staplers is statistically not different from zero and statistically equivalent to zero. That is, robots and staplers are evaluated equivalently on a bipolar valence scale ranging from negative to positive (see Fig 2).

In accordance with our hypotheses, we conducted three paired t-tests with an alpha of .05 to test whether objective ambivalence (H2), subjective ambivalence (H3), and arousal (H4) were higher towards robots than towards staplers. In fact, objective ambivalence was higher towards robots (*M* = 4.31, *SD* = 3.16) than towards staplers (*M* = 2.34, *SD* = 2.82), *t*(44) = 3.18, *p* = .001, with an effect size of *d* = 0.50, indicating a large effect. Also, subjective ambivalence (Cronbach’s α = .86) was higher towards robots (*M* = 5.70, *SD* = 1.49) than towards staplers (*M* = 2.99, *SD* = 1.99), *t*(44) = 9.45, *p* < .001, with an effect size of *d* = 1.41, indicating a large effect. Further, testing the affective consequences of ambivalence, arousal was higher when evaluating robots (*M* = 6.24, *SD* = 1.52) than when evaluating staplers (*M* = 3.53, *SD* = 1.58), *t*(44) = 9.22, *p* < .001, with an effect size of *d* = 1.37, also indicating a large effect.

## Discussion and conclusion

Previous research has shown that in attitude research, the notion of ambivalence is often overlooked and confused with neutrality, although the affective, behavioral, and cognitive consequences associated with both constructs differ greatly [10, 11, 14]. Measuring the positive *and* negative sides of attitudes, along with integrating the opportunity to express evaluative conflict in research on attitudes towards robots, can improve the understanding of attitudes as well as help design adequate interventions aimed at modifying those attitudes. On the one hand, research on attitudes towards robots might benefit from ambivalence research to resolve apparent inconsistencies between attitudes and behavior. On the other hand, social psychological attitude research might benefit from investigating attitudes towards robots, since robots represent exceptional attitude objects. Currently, robots are exceptional in that users hold strong opinions towards them, while simultaneously having relatively little knowledge about and limited experiences with them. In that sense, the psychological experience with robots may be similar to the experience when encountering members of human social outgroups [41]. If so, social robots can be deemed the first non-human humanoid agents towards which humans react with prejudice-like bias. Thus, research on ambivalence in attitudes towards them could provide valuable insights into the reduction of such bias. The specific case of robots provides the unique opportunity to investigate prejudice against stigmatized outgroups in a way that is not confounded by longstanding historical developments as is the case with racism or sexism. Robots are a relatively new outgroup that people have limited knowledge about. Therefore, investigating attitudes towards robots provides an opportunity to understand the impact of outgroup membership per se on attitudes towards outgroups, in the absence of many confounding variables that arise in other contexts related to prejudice.

In the present study we showed that attitudes towards robots were not neutral but rather ambivalent, despite the fact that bipolar measurements had suggested neutral evaluations for both stimuli. Compared to a neutral stimulus, robots evoked high levels of opposing evaluations (i.e., objective ambivalence) and experienced conflict (i.e., subjective ambivalence). This distinction is practically relevant because the consequences associated with ambivalence differ from those of neutral evaluations. We interpret this as evidence for the fact that people apparently feel more conflicted towards robots than towards neutral objects. Consequently, the experienced conflict influences affect. That is, people report higher arousal when evaluating a robot compared to a neutral attitude object. In turn, users with an ambivalent attitude might be tempted to avoid the attitude object due to this arousal, postpone relevant decisions, and respond to different persuasion strategies [6].

When experiencing arousal while making decisions about robots, people could be tempted to avoid the stimulus in the long term, for example by avoiding having a robot at home or avoiding the decision altogether. Furthermore, concerning interventions for improving attitudes towards robots, it could also prove crucial to consider ambivalence. This is because users with neutral attitudes could be interested in more positive information about a robot in order to form an elaborate attitude. Users with ambivalent attitudes might rather benefit from additional information concerning their negative evaluations (e.g., regarding privacy or security concerns or other science fiction inspired fears), and thereby resolve their inner conflict [42]. The literature on ambivalence suggests that a highly ambivalent user might try to avoid the topic, engage in slower decision-making strategies [10], process information selectively [43], orient strongly on peer norms [44] or resolve ambivalence using specific information about the information source [45]. Further research is required in order to determine the specific cognitive consequences of the ambivalent attitudes and arousal observed in this work by including response-time based measures, e.g., tracking mouse movements during decision making, as well as further self-report measures, e.g., concerning information search strategies.

The current research further suggests that the presence of negative evaluations towards robots does not imply the absence of positive evaluations and vice versa. Consequently, when applying widely used scales which measure the negative aspects of robot evaluations, such as the Negative Attitudes toward Robots Scale [46] or Robot Anxiety Scale [47], researchers should be cautious about their interpretations. Highly negative results might reflect negative attitudes—but they might also reflect the negative share of ambivalent attitudes, while neutral results might reflect neutral *or* ambivalent attitudes. Furthermore, for instance, neutral ratings on the Godspeed Questionnaire’s Likeability subscale [48] might reflect neutral *or* ambivalent attitudes. In order to make valid inferences about the attitudes of users, evaluation methods should include the opportunity to express both the negative and positive sides of an attitude, as well as the opportunity to express evaluative conflict. This can be achieved by administering the three-item measure as used in this study [40]. Another possibility is to split items of existing scales into two items each, assessing positive and negative aspects separately. For example, split the Godspeed items concerning likeability, e.g., 1 (nice) to 5 (awful), into two items, respectively, e.g., 1 (not at all nice) to 5 (very nice) and 1 (not at all awful) to 5 (very awful). This way, neutrality (i.e., low values on both items) and ambivalence (i.e., high values on both items) can be differentiated. In the current experiment we specifically instructed participants to think about the positive side of their attitude *while ignoring the negative side* of their attitude and vice versa. However, recent research suggests that this is not always necessary in the assessment of ambivalence [49]. Researchers might decide depending on their items and the research context whether to explicitly instruct participants to partition their judgements.

In sum, research on ambivalence towards robots can help prevent misinterpretations of robot-related attitudes as neutral [cf. 14]. By recognizing ambivalent attitudes, apparent inconsistencies between self-reported attitudes and behavior can be resolved. Prospective research could measure the positive and negative aspects of attitudes towards robots separately and directly assess self-reported conflicting evaluations. Qualitative data can help identify the specific attitude contents that evoke attitudinal conflict. Furthermore, we suggest tailoring interventions or instructions for users of social robots in a way that mitigates user conflict and emotional arousal—for example, by attenuating negative bias by providing concrete information regarding the users specific concerns that helps them resolve attitudinal conflict, or by providing them with the opportunity to consult with peers.

To our knowledge, the present research is the first to directly compare bipolar and multidimensional measurement methods, resulting in concrete recommendations for the measurement of attitudes towards robots. Moreover, we conducted a preregistered experimental study, following an existing paradigm from social psychology. Results were in line with all hypotheses, showing large effects. As a limitation, this study only compares two specific stimuli in a specific student sample—future studies might generalize our results to different robot stimuli within different user samples and consider possible gender effects. Moreover, in this experiment, we used only the word “robot” to investigate attitudes towards robots. We chose the word to capture spontaneous associations with the concept and no pictures were presented to avoid influences of the design on the participants’ attitudes. Currently ongoing research investigates the specific contents of those attitudes, as well as behavioral and cognitive consequences of ambivalence towards robots using various visual stimuli. To conclude, attitudes towards robots are ambivalent and we provide other researchers with the means to distinguish ambivalence from neutrality.

## Supporting information

S1 Data Data and R syntax for the pilot study and the main study. (ZIP)
